# Promising genetic sources for the creation of varieties of durum spring wheat in Western Siberia

**DOI:** 10.18699/VJGB-22-75

**Published:** 2022-11

**Authors:** M.G. Evdokimov, V.S. Yusov, M.N. Kiryakova, L.V. Meshkova, I.V. Pakhotina, D.A. Glushakov

**Affiliations:** Omsk Agrarian Scientific Center, Omsk, Russia; Omsk Agrarian Scientific Center, Omsk, Russia; Omsk Agrarian Scientific Center, Omsk, Russia; Omsk Agrarian Scientific Center, Omsk, Russia; Omsk Agrarian Scientific Center, Omsk, Russia; Omsk Agrarian Scientific Center, Omsk, Russia

**Keywords:** durum wheat, breeding, sample ariety, genotype, yield, grain quality, disease resistance, твердая пшеница, селекция, сорт, образец, генотип, урожайность, качество зерна, устойчивость к болезням

## Abstract

The study, expansion and preservation of the genetic diversity of the source material, and its purposeful use in hybridization is the basis for the creation of adaptive varieties of durum spring wheat that are resistant to biotic and abiotic factors of the environment of Western Siberia. The objects of research were samples of durum spring wheat. Over the years of research (2000–2020), about 3 thousand samples were worked out from the world gene pool of various countries and regions: from the collection of the VIR, the gene pool from Mexico (CIMMYT) within the framework of the agreement and cooperation program (2000–2007), from 2000 to the present time under the program of the Kazakh-Siberian Spring Wheat Breeding Network (KASIB), from other scientif ic institutions of Russia in exchange activities. Using generally accepted techniques, the obtained material was studied for a complex of traits: yield, adaptability, grain quality, resistance to diseases. In the cycle of studying the gene pool from CIMMYT, 50 genotypes were identif ied in terms of yield at the level of the Omskaja jantarnaja standard, 276 grains by test weight, 131 samples by pasta color, 131 samples by resistance to hard smut, and 112 by resistance to powdery mildew. Almost all samples were not affected by leaf rust. The study set showed high sensitivity to extreme conditions and most forms of interest in quality and disease resistance were low-productive in our environment. In KASIB nurseries, 29 samples were identif ied in terms of yield and adaptability, 29 samples in terms of grain quality, 21 in terms of resistance to diseases, including 8 resistant to stem rust. In the set of varieties received from the VIR, 15 genotypes were adaptive, 16 had high grain quality, 11 were resistant to stem rust. In the breeding material, 17 samples of the local population resistant to stem rust (6 of them were comprehensively resistant) and 25 race-resistant to Ug99 were identif ied. The genotypes identif ied as a result of research are of interest as sources of valuable traits.

## Abstract (ru)

Изучение, расширение и сохранение генетического разнообразия исходного материала и его целенаправленное использование в гибридизации являются основой для создания адаптивных сортов твердой яровой пшеницы, устойчивых к биотическим и абиотическим факторам среды Западной Сибири. Объектами исследований служили образцы твердой яровой пшеницы. За годы исследований (2000–2020) проведен скрининг более 3000 образцов из мирового генофонда разных стран и регионов: из коллекции ВИР, банка генетических ресурсов CIMMYT (Мексика) в рамках договора и программы сотрудничества (2000–2007 гг.), с 2000 г. по настоящее время по программе Казахстанско-Сибирской сети по улучшению яровой пшеницы (КАСИБ), из других научных учреждений России в порядке обмена селекционным материалом. Полученный материал был изучен с использованием общепринятых методик по комплексу признаков: урожайности, адаптивности, качеству зерна, устойчивости к болезням. При исследовании в 2000–2007 гг. генофонда из CIMMYT по урожайности на уровне стандарта Омская янтарная было выделено 50 генотипов, по натуре зерна – 276, по цвету макарон – 131, по устойчивости к твердой головне – 131, мучнистой росе – 112. Почти все образцы не поражались бурой ржавчиной. Изученный набор показал высокую чувствительность к экстремальным условиям; большинство форм, представляющих интерес по качеству и устойчивости к болезням, были низкопродуктивными в наших условиях. В питомниках КАСИБ по урожайности и адаптивности выделено 29 образцов, по качеству зерна – 29, устойчивости к болезням – 21, в том числе к стеблевой ржавчине – 8. В наборе сортов, поступивших из ВИР, выявлено 15 адаптивных генотипов, 16 – с высоким качеством зерна, 11 – устойчивых к стеблевой ржавчине. При оценке селекционного материала выделено 17 образцов, устойчивых к стеблевой ржавчине местной популяции (6 из них комплексно устойчивы к бурой, стеблевой ржавчине, мучнистой росе), и 25 резистентных к расе Ug99. Выделенные в результате исследований генотипы представляют интерес как источники ценных признаков.

## Introduction

Hybridization with targeted selection of parental forms remains one of the most important ways to create durum wheat varieties. Therefore, the study of the source material is the main factor in successful breeding. The doctrine of the source material was developed by K.A. Flaksberger (1934), N.I. Vavilov (1935) and was further developed in the works of many researchers.

The main bank of genetic resources is the N.I. Vavilov All-Russian Institute of Plant Genetic Resources (VIR) with its branches and bases in various climatic zones of the country, the number of which, unfortunately, has greatly decreased in recent years (Lyapunova, Andreeva, 2020). From 2000 to 2007, a large number of samples was received from the International Center for the Improvement of Maize and Wheat (CIMMYT, Mexico) within the collaboration under the agreement and cooperation program; from 2000 to the present time, samples have been received under the program of the Kazakh-Siberian Network for the Improve- ment of Spring Durum Wheat (KASIB). At the same time, the basis for the creation of varieties is the breeding material obtained with the involvement of samples from CIMMYT and exposed to natural selection in local soil and climatic conditions

In recent years, interest in local and ancient varieties of durum wheat has increased (Pagnotta et al., 2005; Kan et al., 2014; Peneva, Lyapunova, 2020), as they are characterized by unique features and, above all, resistance to a number of adverse environmental factors that have a major impact on plant survival, and to some races of local populations of fungal and bacterial diseases.

In the last century, many works were devoted to the evaluation of the source material carried out in the condi- tions of Siberia, the Volga region, Ukraine, Kazakhstan, Uzbekistan and other regions of the former Soviet Union (Evdokimov, 2006). In recent years, the trend has been reflected in the works of domestic scientists engaged in the directions of selection to increase yields, adaptability, grain quality and disease resistance (Ziborov, Rozova, 2012; Evdokimov et al., 2017; Malchikov et al., 2018; Mukhitov, Timoshenkova, 2018; Samofalova et al., 2018; Dorokhova, Kopus, 2020; Rozova et al., 2020; Malchikov, Myasnikova, 2021;Yusov et al., 2021).

The need to study the collection material in Siberian conditions lies in the fact that the behavior of the genotype in different environmental conditions is far from the same. At the same time, the study of the source material should be carried out taking into account the main directions of breeding: further increase in yield and adaptive potential, quality of grain and pasta, resistance to diseases and stabi- lity of agronomically important traits. For the Omsk region, with sharp fluctuations in meteorological factors during the growing season and by year, such stability is of paramount importance.

Preservation, study and replenishment of the gene pool with new forms is relevant in the purposeful screening of source material in breeding programs (Likhenko et al., 2014). This will make it possible to make a certain contribu- tion to the creation of varieties that meet the requirements of agricultural production and the implementation of the scientific program “Bread of Russia” in 2022–2027, which is aimed at accelerating, stabilizing the selection process and, ultimately, ensuring the country’s food security.

The main aim is to identify promising sources of agro- nomically important traits for the creation of varieties of durum spring wheat in the conditions of Western Siberia

## Materials and methods

The objects of research were samples from the VIR collec- tion. From 2000 to 2007, a large number of samples was received from CIMMYT within the collaboration under the agreement and cooperation program, from 2000 to the pre- sent time samples have been received through the Kazakh- Siberian Network (KASIB). In recent years, varieties and breeding material have been obtained from other scientific institutions of Russia (Altai Research Institute of Agricul- tural Sciences, Samara Research Institute of Agricultural Sciences, Research Institute of Agricultural Sciences of the South-East, Voronezh FASC named after Dokuchaev) as part of an exchange.

The principle of the approach to the development of the material was as follows: after the first year of study, samples with low values for a set of indicators were rejected, and the selected genotypes were further tested in the second year. For three years, only promising samples were tested. The number of genotypes studied was more than 3 thousand.

A significant part of the gene pool was from North Ame rica – Mexico, USA, Canada; Russia, CIS countries – Ka- zakhstan, Azerbaijan; Ukraine; European countries – Italy, Spain, Portugal, France; a small number of samples came from the Middle East – Turkey, Israel, Yemen; Central, East and South Asia – Iran, China, India; North Africa – Algeria, Morocco, Tunisia, Ethiopia; South America – Chili (Table 1). The bulk of the material from the North American continent came from Mexico (CIMMYT). In 2000–2007, with an annual intake of 3 nurseries (IDYN – International Durum Yield Nursery, EDUIT – Elite Durum Unrepricield Yield Treals, IDSN – International Durum Screening Nur sery), the total volume was 2711 samples. Under the KASIB program, 210 genotypes were studied, and 186 genotypes were studied from the VIR revenues. In addition, samples were studied at the final stages of the selection process (preliminary and competitive variety testing).

**Table 1.**
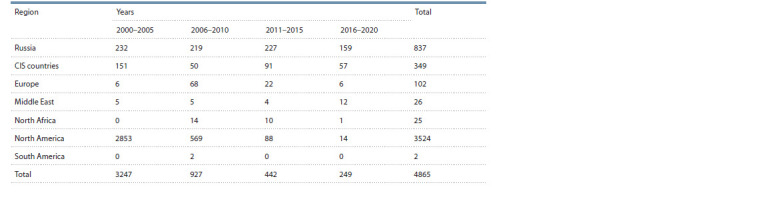
Volume of study of the collection material of spring durum wheat (2000–2020)

To study the gene pool of durum wheat, a collection nur sery was annually laid in the breeding stationary of the durum wheat breeding laboratory in accordance with the guidelines of the VIR (Merezhko et al., 1999), as well as a nursery of competitive and environmental tests according to the GSU methodology (Methodology of State Variety Testing..., 2019). In 2000–2008, the Omskaya jantarnaya variety was used as a standard, and since 2009 an additional standard has been introduced – the Jemthujina Sibiri.

Sowing was carried out on plots with an accounting area of 2–3 m2 (collection), 10 m2 (competitive, environmental test) in 2–4 repetitions with the SSFC7 planter. Harvesting of plots was carried out by the combine HEGE 125.

Indicators of the test weight of grain, pasta properties, sus- ceptibility to major diseases were determined by generally accepted methods (Kolmakov, 2007; Koishybayev, 2018). To assess susceptibility, the CIMMYT scale was used: 0 – im- mune, there are no signs of the disease; R – stable, chlorous spots are formed, occupying up to 5–10 % of the leaf surface (on the Stekman scale, 1 point); MR – medium resistance, pustules are small, there are chlorotic zones occupying no more than 10–25 % (2 points); MS – medium susceptibility, pustules are small, occupy up to 40–50 % of the leaf surface (3 points); S – high susceptibility, pustules are large, occupy up to 50–100 % of the leaf surface (4 points).

Mathematical processing of the results was carried out according to B.A. Dospekhov (2012) using a package of applied statistical programs Microsoft Excel. The parame- ters of ecological plasticity were calculated according to S.A. Eberhart, W.A. Russel in the presentation of V.A. Zykin and co-authors (Zykin et al., 2011). Analysis of principal components (Principal compatible analysis, PCA) was car- ried out using the R version 4.0.3 package

## Results and discussion

Yield and adaptability

The studied samples in nurseries from CIMMYT in terms of yield were significantly inferior to the Omskaya jantar- naya standard. The average yield in nurseries ranged from 51.6 to 87.5 %. The number of genotypes at or above the standard level in kennels 32 IDYN, 37 IDYN, 38 IDYN, 35 EDUYT was 1–2, in 33 IDYN, 34 IDYN, 36 IDYN – 3–4, in 30 EDUYT, 34 EDUYT, 36 EDUYT – 5–6, and only in 32 EDUYT – 18 samples. In nurseries 35 IDYN, 31 EDUYT, 33 EDUYT, not a single sample formed a yield at the level of the Omskaya jantarnaya variety.

In terms of yield and adaptability in these nurseries, Anade 1/Tarro 1//Lican (32 IDYN), Nehama 15/Brisina 2// Plata 9 deserve attention (30 EDUYT), SN Turk MI83-84/ Nigris5; GA//2* Chen/Altar 84; Cado/Boomer 33; Dip- per 2/Bushen 3; Himan 9/Lotus 1; Crake 10/Rissa; Chen/ Altar 84/3/Hui//Poc//Bub/Rufo/4/Fnfoot (32 EDUYT), Cndo/Vee//7*Plata 8/3/Plata_L/Snm//Plata 9; Vanrrikse 14/ Plata 6//Green 17; Plata 22/3/Magh 72/D67.2//FGO (34 EDUYT), Arment//Srn_3/Nigris 4/3/Canelo 9.1 (35 EDUYT), Minimus_6/Plata 16; Ajaia_16//Hora/JRO (36 EDUYT). Among those presented in Table 2, 15 geno- types combine yield with high nature, 5 with the color of pasta, 8 with resistance to hard smut, 6 to powdery mildew, 25 to leaf rust.

**Table 2.**
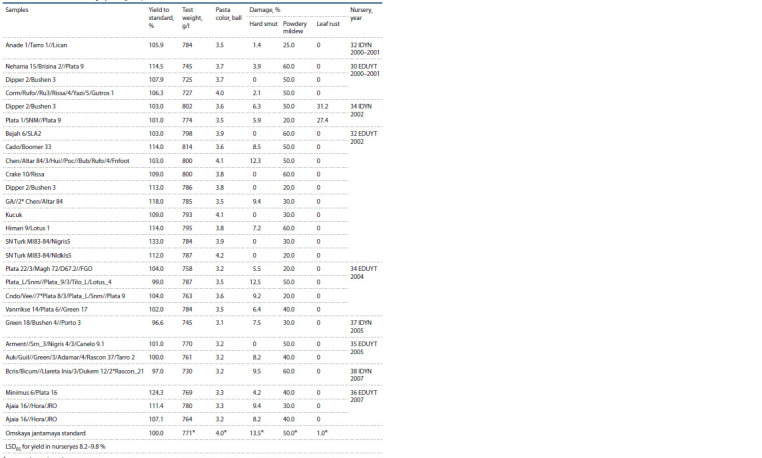
Characteristics of the high-yielding samples from CIMMYT nurseries * Average data on kennels are given.

Among the studied varieties of the VIR collection in 2000–2003, the following were identified in terms of yield: k-59881, k-60388, k-60364, k-60366, k-60413, k-61303, the following samples had an advantage and the color of the pasta: k-59881, k-60388, k-60364. All these specimens were resistant to lodging, due to the optimal ratio of anatomical features of the stem. In the 2007–2008 cycle, shortstemmed samples from Europe, the United States and Canada were tested. Due to the shortened lower internodes, they are highly resistant to lodging, their disadvantages are low drought resistance and yield. However, 4 samples k-62658, k-63126, k-63160, k-64353 formed a yield at the level of the Omskaya jantarnaya standard and above (with an increase of 1–18 %), but they do not represent breeding value in terms of grain quality (Table 3).

**Table 3.**
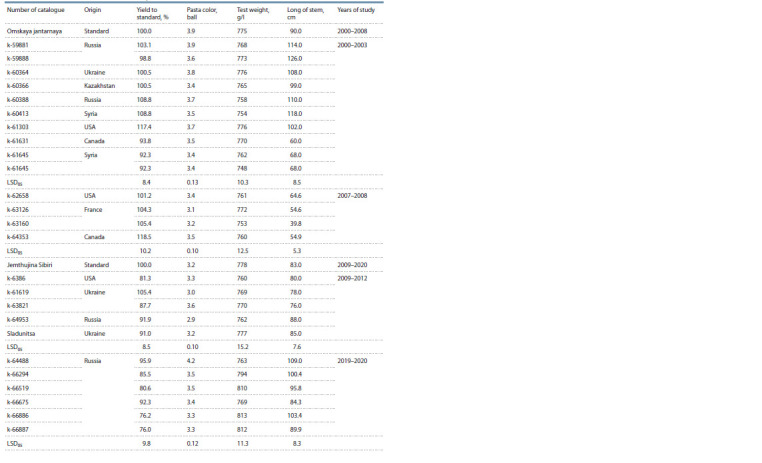
Characteristics of the highest productivity samples from the VIR collection

In 2009–2012, out of 62 genotypes, only one (k61619) formed a yield above the Jemthujina Sibiri standard by 18.5 %, but by the test weight of the grain and the color of pasta, the indicators were low. When studied in 2019–2020, the most productive forms had a yield of 92–96 % in relation to the yield level of the Jemthujina Sibiri standard – k64488, k-66675.

The Kazakh-Siberian Spring Wheat Improvement Net- work (KASIB), established in 1999, provides for the ex- change of genetic material and the testing of samples over a vast territory of Russia and the Republic of Kazakhstan (43–55° N, 55–85° E) with an annual precipitation range of 250–500 mm. The main advantage of this project is that within one year when tested in different ecological points, and there are 6–8 of them for durum wheat, it is possible to evaluate genotypes by a complex of traits: adaptability, drought resistance, stability and purposeful inclusion of them in the breeding process as sources of the main economically valuable traits.

Table 4 presents the most productive varieties and lines in the conditions of Omsk that formed a high average yield for all points of variety testing of the KASIB network, created in Russia and Kazakhstan. Among them, 18 have a rank of 1–3 in terms of average yield and are adaptive forms. According to the Eberhart–Russell test, genotypes 242.93, G.43088, (Каrabalyk Agricultural Experimental Station, AES), G.97491, Omskiy corall (Omsk Agrarian Scientific Center, ASC), G.748 (FASCA) are intense – bi = 1.24–1.89, extensive include Omskaya jantarnaya (Omsk ASC), Karga- la 3, Kargala 30, Kargala 69 (Aktobe AES) – bi = 0.55 0.89. Variance deviations from the regression line (σ2di) indicate that they form a stable yield (see Table 4).

**Table 4.**
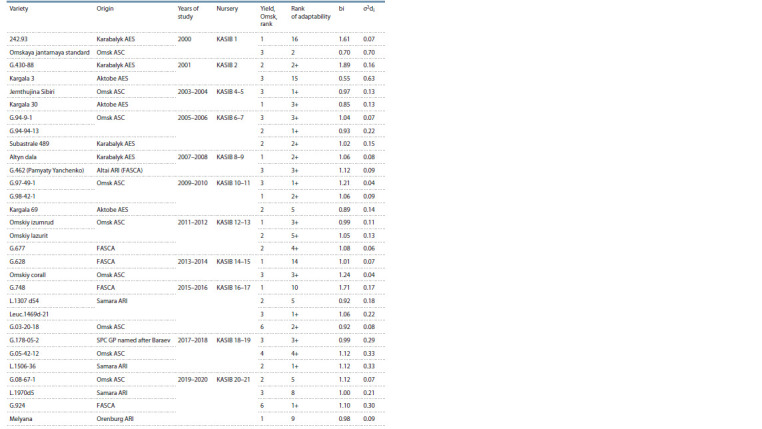
Sources of high productivity and adaptability from KASIB nurseries Notе. G – hordeiforme, Leuc. – leucurum, L – line. AES – Agricultural Experimental Station; ASC – Agrarian Scientific Center; ARI – Agricultural Research Institute; FASCA – Federal Altai Scientific Center for Agrobiotechnology; SPC GP named after Baraev – Scientific and Production Center of Grain Farming named after A.I. Barayev.

Grain quality and pasta properties

Among the CIMMYT material by test weight of grain 276 samples were allocated, by the color of pasta – 131 sam- ples. The studied set showed a high response to extreme stressors and most of the forms of interest in grain quality and resistance to diseases in the conditions of the Omsk region were low-productive. Therefore, 56 genotypes are of paramount importance in terms of grain and pasta qua- lity. By test weight grains and pasta quality in CIMMYT nurseries deserve attention those in 32 IDYN – Topdy 18/ Focha 1//Altar 84 (test weight 807 g/l, 4.1), Dipper 2/ Bushen 3, Rascon 37/2* Tarro 2; in 30 EDUYT – Ajata/ Bichena, Yavaus/Tez//Altar 84, Wizza 23/Cona, Fulvous 1/ Meowl 13, Dusky 12/Bushen 4, Cham 3/Comdk//Ajata; in 34 IDYN – Dipper 2/Bushen 3, Yel/Bar/3/Garza/AFN, Rascon 39/Tilo 1; in 32 EDUYT – Chen/Altar 4/3/Hui/..., Eupoda 3/Suv 2//Minimus, Kucuk, SN Turk MI83-84/ Nldkls5; in 36 IDYN – Tarro 1/2* Yual 1/Ajata 13, Duck 2// Cham 3/3/Canelo 9; in 34 EDUYT – samples Plata 1/SND// Plata 9, SN Sturk M 183-84503/Lotus 14, GS/CRA/SBA 81; in 38 IDYN – 1A.1D5+10/2*WB881, Skest/Krm//Sla/3/...; in 36 EDUYT – Ajaia 12/F3Local, Stot//Altar 84/ALD, Rascon 21/3/Mque. A detailed description of the above sources is presented in the Suppl. Material 1^1^.

Supplementary Materials are available in the online version of the paper: http://vavilov.elpub.ru/jour/manager/files/Suppl_Evdokimov_27_7.pdf


By test weight grains and the color of the pasta, of interest as sources are the samples from the VIR: k-59881, k-59889, k60388, k60364, k6386; by nature – k63281, Sladunitsa; according to color estimates, pasta – k61117, k62657, k-64353, k-64355, k-64354, k-17985, k-60410.

In KASIB nurseries, grains are valuable by test weight – Kargala 1538 (Аktobe Agricultural Experimental Station), Altyn Dala, Sharifa (Karabalyk Agricultural Experimental Station), Lan (Kazakh Research Institute of Agriculture and Plant Growing), G.178-05-2, Line 250-06-14 (SPC GP named after Baraev), G.94-24-12, G.96-160-8 (Omskaya stepnaya), Omskiy izumrud, G.98-42-5 (Omskiy zircon), G.00-96-8 (Omskiy lazurit), G.04-85-4 (Omskiy corall), 1 Supplementary Materials 1–4 are available in the online version of the paper: http://vavilov.elpub.ru/jour/manager/files/Suppl_Evdokimov_27_7.pdf G.00-178-4 (Omskaya birjusa), G.05-42-12, G.08-67-1 (Omsk ASC), G.677, G.829, G.864 (FASCA), Line 653d-44, L.1469d-21, G.1591-21, Line 1970d-5, Line 2021d-1 (Sa- mara ARI), Luch 25, Line D-2165 (Research Institute of Agricultural Sciences of the South-East), Melyana (Oren- burg ARI). According to the pasta color assessment, Omsk varieties and lines are allocated – G.942412, Omskaya stepnaya, Omskiy zircon, Omskiy lazurit, G.05-42-12, Omskiy izumrud, G.08671, Altai – G.677, G.864, Sama ra ARI – Line 653d44, Saratov – Luch 25, Kazakhstan – G.178-05-2 (Suppl. Material 2). Of great importance are the genotypes of Omskiy zircon, Omskiy lazurit, G.05-42-12, G.864, Line 653d-44, forming a grain with a high test weight and color of pasta.

Resistance to biotic factors

Currently, one of the directions of ecological farming is the creation of immune varieties for pesticide-free technologies. Selection for disease resistance is a rather time-consuming and complex aspect since each pathogen has an extensive set of physiological races and evolves quite quickly, often ahead of the selection process of the new variety. Therefore, the search for new resistance genes is one of the most important in the strategy of plant protection

In CIMMYT nurseries, for resistance to hard smut, 131 genotypes (0–1.0 %) were revealed, to powdery mil- dew – 112 (6–7 points). Almost all samples were not affected by leaf rust. Among the samples that have an advantage in other parameters, 54 were resistant to hard smut, leaf rust, 38 – to powdery mildew. The most interesting are the forms that are resistant to 2–3 diseases. These include Srn 2// Yavaus/Hui/3/ (36 IDYN), Malmuk 1/Serrator, Kucuk 2/ Pata 2 (34 EDUYT) that showed immunity to hard smut, powdery mildew, and leaf rust (damage grade 0). Of great- est interest are genotypes that combine resistance with high rates of grain test weight and pasta color. First of all, we should highlight the samples Dipper 2/Bushen 3, Chen/ Altar 84/3/Hui//Poc//Bub/Rufo/4/Fnfoot (32 IDYN); Lhnke/ Rascon//Cona, Fulvous 1/Mfowl 13/3/Stot//Altar 84/Ald (30 EDUYT); Rascon 39/Tilo 1, Yel/Bar/3/Garza/AFN/ (34 IDYN); Srn 2//Yavaus/Hui/3/, Cndo/Primadur//Hai (36 IDYN); Ajaia 4/Yebas, SN Turk MI83-84, Tarro l/Yuan, SN Turk MI83-84 03/Lotus, Plata 20/Fillo// (34 EDUYT) (Suppl. Material 3). Genotypes Fulvous 1/Meowl 13// Altar 84, Chen//Altar 84... carry resistance genes Lr23, Sr B, Sr E transmitted from the cultivar Altar 84 (McIntosh et al., 2008).

All the forms distinguished in terms of grain quality and disease resistance were actively involved in the breeding process. Only in the period from 2001 to 2006, with the participation of Mexican forms, crosses were carried out on 215 hybrid combinations. The share of hybrid combinations with Mexican samples in these years was 31.6–53.4 %. In 2007, a selection was made from the hybrid combination Omskaya jantarnaya//Pod 11/Yazi (31 EDUYT), which, subsequently, in 2018 was transferred to the State Test under the name of the ‘Omskiy corall’ variety, and included in the State Register of Breeding Achievements in 2021. However, these lines are of interest as a starting material for further breeding process.

In Western Siberia, leaf rust, hard and dusty smut, pow- dery mildew were common among the diseases, and until recently there was no manifestation of stem rust. The first foci on spring soft wheat stem rust were discovered in 2007, from 2008 to 2014 it was observed annually to varying degrees, but the damage did not exceed 50 %, and epiphyto tics of stem rust arose starting from 2015 (Rosseeva et al., 2019). In subsequent years, stem rust on durum wheat ap- peared regularly with a degree of damage from 70 to 100 % (Gultyaeva et al., 2020; Yusov et al., 2021). In recent years, epiphytotics of wheat stem rust have been noted in the north- ern regions of Kazakhstan and in the territories adjacent to the Omsk region of Russia. It was noted that the increase in the frequency of epiphytotics of stem rust is associated with the emergence of new virulent races of the causative agent of the disease and the cultivation of susceptible varieties of wheat (Rsaliev A.S., Rsaliev Sh.S., 2018).

The results of the evaluation of isogenic lines from the CIMMYT International Stem Rust Trap Nursery (ISRTN) in the field in 2019 with maximum damage showed that genes Sr23 (Exchange), Sr25 (Agatha(CI14048)/9*NMPG- 6DK16), Sr31 (Seri 82), Sr38 (Trident) (degree of damage 10 %, infection type R–MR) are effective against the local stem rust population. Genes Sr21 (Einkorn), Sr26 (Eagle Sr26), Sr39 (RL 5711), Sr40 (RL 6087); pyramids of genes Sr6, Sr24, Sr36, 1RS-Am (Fleming) and Sr7a, Sr12, Sr6 (Chris) inhibit the damage (up to 20 %). The remaining lines were affected by 30–80 %, with the type of infection MS–S (Table 5). The susceptibility standard had a lesion rate of 90 % (infection type S). The high efficiency of the Sr31, Sr38, Sr40 genes was previously identified in the conditions of Omsk by V.P. Shamanin and colleagues (2020). It should be noted that the effectiveness of genes Sr21, Sr31 in differ- ent varieties was different. The Seri 82 variety showed re- sistance to the population, and the line (Benno)/8*L MPG-8 DK42, also carrying the Sr31 gene, was affected. A similar picture was observed in the effectiveness of the Sr21 gene, which was previously noted by L.P. Rosseeva and colleagues (Rosseeva et al., 2017).

**Table 5.**
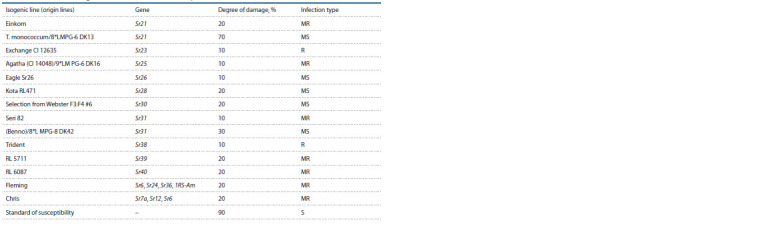
Characteristics of isogenic lines on resistance to stem rust, 2019

The racial composition of stem rust populations varies considerably from region to region. In addition, the biotype composition of races is not of the same type. According to M.S. Hovmøller (2017), a comparative analysis of the TTTTF race isolated in Omsk differs significantly from that of the Sicilian race. This explains the differences in the efficiency of Sr genes in territorial and temporal space (Sochalova, Likhenko, 2013; Rosseeva et al., 2017).

Over the 19 years of the KASIB program’s existence, 210 samples have been studied. As sources for resistance to the local population of stem rust, the following varieties have been distinguished: G.03-20-18, Omskaya jantarnaya, Omskiy izumrud, G.04-85-4 (Omskiy corall), G.05-42-12, G.08-67-1, G.08-107-5 (Omsk ASC), Kargala 28, Kargala 303, Kargala 1412, Kargala 1514, Kargala 1516/06 (Aktobe AES); Lines 688d-4, 1591d-21, 1560d-18 (Samara ARI); Durum 49, G.69-08-5, G.178-05-2, Line 250-06-14 (SPC GP named after Barayev); Line No. 9 from Karabalyk AES (Yusov et al., 2018)

Among the VIR samples, the breeding value for resistance to stem rust is: k-6386, k-6662, k-46983, k-60410, Iride, k-65353, k-65733, k-65734.

**Fig. 1.**
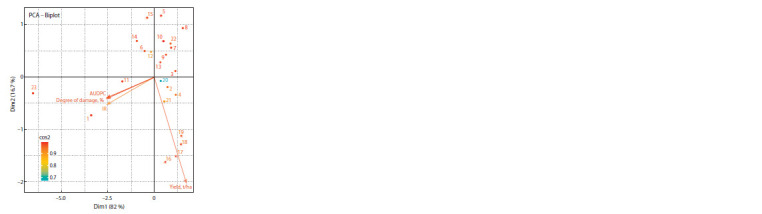
Distribution of varieties and lines of durum wheat in the plane of the main components by the degree of stem rust damage in the field in 2019–2020. 1, Jemthujina Sibiri; 2, Omskiy izumrud; 3, Omskiy corall; 4, Triada; 5, Odysseo; 6, Soyana; 7, G.250-06-14; 8, Line 1927d; 9, G.07-115-1v; 10, G.08- 76-1; 11, G.08- 107-5; 12, G.0-68-1; 13, G.09-122-1; 14, G.10-32-4; 15, G.10- 33- 4; 16, G.11-48-12; 17, G.12-9-3; 18, G.16-8-2; 19, G.16-8-4; 20, G.16-8-5; 21, G.16- 13-2; 22, G.16-13-4.

A comparative study of the varieties of ecological testing and lines created in the Omsk ASC showed that there are resistance forms, but the genetic control of resistance in them is not due to oligogenes (genes of vertical resistance). Varieties Omskiy izumrud, Omskiy corall, Triada, Odys- seo, G.250-06-14, Lines 1927d, G.07-115-1v, G.08-76-1, G.09-122-1, G.12-9-3, Lines 2016-8-2, 2016-8-4, 2016-13-4; in accordance with the classification of A.A. Makarov and colleagues (Makarov et al., 2003) these are genotypes with high racially specific resistance, the resistance index of which is, on average, for 2019–2020, 0.21–0.40. They have a delayed development of the disease, and as a result, a low value of the AUDPC (area under the disease progress curve), which ranges from 542 in the Omskiy corall to 696 c.u. (conventional units) in the Omskiy izumrud, with the value of the standard variety Jemthujina Sibiri being (1626), susceptibility standards being (2230–2873 c.u.). The mini- mum value of the degree of stem rust damage (16.7 %) was noted in the Line of 1927d (Fig. 1). Their yields were above standard. These varieties have a pronounced nonspecific resistance, which is expressed by the delayed development of the disease and can persist for a long time. The genotypes of Soyana, G.08-107-5, G.09-68-1, G.10-32-4, G.10-33-4, G.11-48-12, G.16-8-5, G.16-13-2 have moderate racial- specific resistance.

**Fig. 2.**
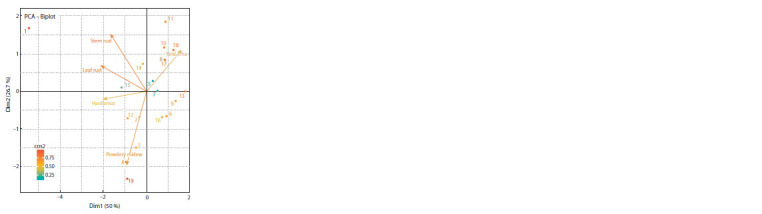
Analysis of the main components of the main agronomically important traits of competitive and preliminary variety trial of spring durum wheat (in average for 2018–2020). 1, Jemthujina Sibiri; 2, Omskiy izumrud; 3, G.08-67-1; 4, G.09-122-1; 5, G.09- 73- 1; 6, G.10-32-3-1; 7, G.10-32-12; 8, G.10-63-1; 9, G.10-71-3; 10, G.11- 45- 2; 11, G.11- 46-3; 12, G.11-97-3; 13, G.11-98-3; 14, G.12-11-1; 15, G.12- 12-2; 16, G.11- 92-2; 17, G.11-75-1; 18, G.12-31-1; 19, G 13-18-3.

The problem of resistance to diseases, including stem rust, has always been given special attention in the breed- ing programs of Omsk ASC, therefore, at present, varieties, promising samples and lines that are of interest primarily as sources of resistance to this pathogen have been created. At the final stages of the breeding process, 15 genotypes resistant to leaf rust, 11 to stem rust, 8 to hard smut, 10 to powdery mildew were identified. Highly productive breed- ing lines with a yield of more than 5.0 t/ha (Jemthujina Sibiri standard – 4.5 t/ha), with complex resistance to 3–4 diseases have been created: G.10-32-3-1, G.10-63-1, G.10-71-3, G.11-98-3, G.11-75-1, G.12-31-1 (Fig. 2).

Along with this, there is a danger and a threat of penetra- tion from the countries of the Middle East and Central Asia of the malicious race of stem rust Ug99, which was first discovered on the African continent in Uganda and named after the place of its first discovery (Shamanin et al., 2015). A cause for concern is the TTKSK pathotype, which has high virulent properties and overcomes the effectiveness of many wheat resistance genes, including the Sr31 gene (Singh et al., 2015). The effectivity of Sr9e durum wheat genes in Kronos (Li et al., 2021), Sr13 in Cirilla (Laido et al., 2015) and Fielder (Zhang et al., 2017) in Africa and Kronos, Kofa, Medora, Scepter varieties in Canada (Simmons et al., 2011) have been shown. Effective under Canadian conditions, genes Sr8 and Sr14 have been identified in grade A9919 BY5C (Kumar et al., 2021).

In accordance with the program of international coope- ration under the auspices of CIMMYT, breeding material created in the Omsk ASC, as well as samples and lines of KASIB, were sent to Kenya for evaluation in different years. In the kennels of KASIB, 7 genotypes showed resistance to the Ug99 race: Durum 49, Lavina (SPC GP named after Barayev), G.950/99 (Karabalyk AES), G.748 (FASCA), L.1307d-54 (Samara ARI), Omskiy izumrud, Omskiy lazu- rit, G.11-77-3 (Omsk ASC). When evaluating the breeding nursery, 27 numbers showed resistance to the Ug99 race. Among the immune forms are G.08-55-5, G.08-94-3, G.12-17-2 (Table 6).

**Table 6.**
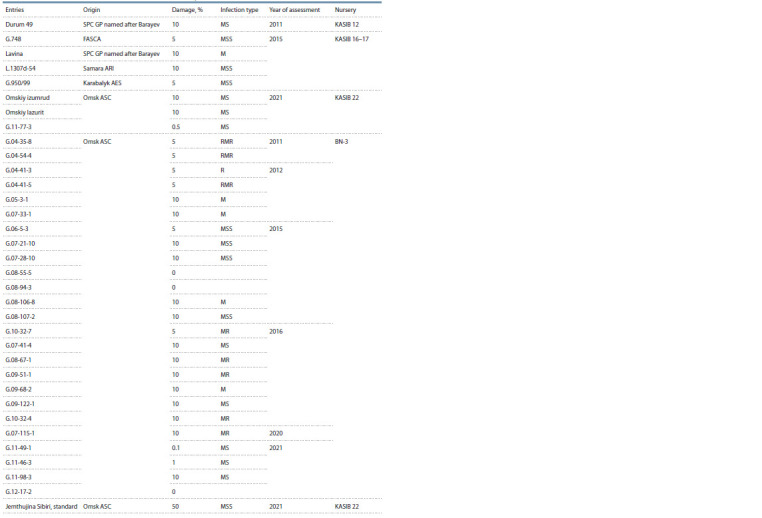
Entries resistant to stem rust race Ug99 (assessment in Kenya)

## Conclusion

Based on the studies conducted in 2000–2021, when studying the gene pool of durum wheat from CIMMYT, 50 genotypes were identified at the level of the Omskaya jantarnaya standard in terms of yield, 276 grains by test weight, 131 samples by pasta color, 131 samples in terms of resistance to hard smut, and 112 samples to powdery mildew. Almost all samples were not affected by leaf rust. The studied set of samples are of interest due to their grain quality and disease resistance but have low productivity in the conditions of the southern forest-steppe of Western Siberia. 56 genotypes have been identified for resistance to hard smut, 54 – to leaf rust, 38 – to powdery mildew, in combination with other valuable features

In KASIB nurseries, 29 samples have been selected for high yield and adaptability, 29 for grain quality, 21 for disease resistance, including 8 for resistance to stem rust. Among the varieties from the VIR collection, there are 15 adaptive genotypes, 16 with high grain quality, and 11 resistant to stem rust.

In the conditions of the Omsk region, effective genes for resistance to the local population of stem rust are Sr23, Sr25, Sr26, Sr31, Sr38. Sr39, Sr40 genes; pyramids of genes Sr6, Sr24, Sr36, 1RS-Am (Fleming) and Sr7a, Sr12, Sr6 (Chris) restrain the damage (up to 20 %).

A new breeding material has been created that combines complex resistance to leaf, stem rust, hard smut, powdery mildew with high yields and good grain quality. When evaluating the breeding material, 17 numbers resistant to the local population of stem rust (6 of them have complex resistance) and 25 raceresistant to Ug99 were identified.

The genotypes identified as a result of research are of interest as sources of valuable traits. Part of the studied material is included in the scientific program of the “Bread of Russia”.

The studied gene pool of durum wheat, which includes a large set of varietal samples of various ecological and geographical origin, will contribute to the purposeful se- lection of parent pairs, in accordance with the principles of geographical remoteness and genetic divergence, developed by N.I. Vavilov (1935), which are still relevant at the pre- sent time.

## Conflict of interest

The authors declare no conflict of interest.
